# Framework of metal insertion in the projection domain for image quality optimization in interventional computed tomography

**DOI:** 10.1117/1.JMI.9.3.035001

**Published:** 2022-06-17

**Authors:** Liqiang Ren, Andrea Ferrero, Christopher Favazza

**Affiliations:** Mayo Clinic, Department of Radiology, Rochester, Minnesota, United States

**Keywords:** metal insertion, interventional computed tomography, image quality optimization, ablation probes, metal artifact

## Abstract

**Purpose:**

This work aims to develop a framework to accurately and efficiently simulate metallic objects used during interventional oncology (IO) procedures and their artifacts in computed tomography (CT) images of different body regions.

**Approach:**

A metal insertion framework based on an existing lesion insertion tool was developed. Noise and beam hardening models were incorporated into the model and validated by comparing images of real and artificially inserted metallic rods of known material composition and dimensions. The framework was further validated by inserting ablation probes into a water phantom and comparing image appearance to scans of real probes at matching locations in the phantom. Finally, a comprehensive library of metallic probes used in our IO practice was generated and a graphical user interface was built to efficiently insert any number of probes at arbitrary positions in patient CT data, including projection and image domain insertions.

**Results:**

Metallic rod experiments demonstrated that noise and beam hardening were properly modeled. Phantom and patient data with virtually inserted probes demonstrated similar artifact appearance and magnitude compared with real probes. The developed user interface resulted in accurately co-registered virtual probes both with and without accompanying artifacts from projection and image domain insertions, respectively.

**Conclusions:**

The developed metal insertion framework successfully replicates metallic object and artifact appearance with projection domain insertions and provides corresponding artifact-free images with the metallic object in the identical location through image domain insertion. This framework has potential to generate robust training libraries for deep learning algorithms and facilitate image quality optimization in interventional CT.

## 1 Introduction

Percutaneous ablation has achieved broad clinical applications in locally treating metastatic lesions over the past decade.^1^ In part due to wide availability and excellent ability to delineate tumors from surrounding materials, computed tomography (CT) image guidance is routinely relied upon for treatment planning, intraprocedural guidance and monitoring, and post-treatment assessment of interventional oncology (IO) procedures.^2^^,^^3^ One drawback of CT imaging during these ablation procedures, however, is the potentially severe artifacts introduced by the metallic probes and devices used for treatment. These metal artifacts degrade the image quality in the anatomical region of interest (ROI). As a result, the interventional radiologist’s confidence in placement accuracy of the probe(s) relative to the tumor and sensitive tissues may be substantially reduced.^4^^,^^5^ Additionally, probe-induced artifacts may limit treatment zone evaluation during treatment monitoring. The artifacts generated by these probes vary greatly with their type, number, and relative orientation. As a result, CT protocol optimization for interventional procedures has been shown to be challenging and complex.^5^

Although all CT manufacturers offer solutions to mitigate the impact of metal artifacts on image quality, currently available strategies have limited effectiveness for CT-guided IO procedures. This is in part due to the uniquely challenging artifacts presented in the interventional CT images caused by metallic probes and their mutual interactions as compared with more common metallic implants that conventional artifact reduction algorithms are designed to reduce. For example, one commercial product, metal artifact reduction for orthopedic implants (O-MAR) was optimized to correct for orthopedic metal implants that are embedded in normal tissue; when the metal protrudes beyond the skin boundary, the O-MAR algorithm could erroneously cause the extension of the skin boundary.^6^ O-MAR, therefore, should not be employed when metal extends beyond the skin; however, this is the case encountered in IO procedures. Other strategies for MAR such as virtual monoenergetic imaging based on dual energy or spectral techniques, high kV acquisitions, and wide window presentations are undesirable and inadequate as CT images acquired during IO procedures are typically time-sensitive and require sufficient soft tissue contrast. In general, MAR algorithms vary in performance with limited generalizability,^7^^–^^9^ and there is currently no universally applicable commercial tool available for IO procedures. Thus, a tool to facilitate efficient and systematic investigations of all variable combinations on image quality and CT protocol optimization for interventional procedures would likely provide great benefits to individual practices and the field at large.

The purpose of this work is to develop a framework for metallic object insertion in the projection domain and tailor it for interventional CT applications through detailed modeling of ablation probes for image quality optimization. By accurately simulating the appearance of metallic objects and their induced image artifacts regardless of their number, type, and relative orientation, such a metal insertion framework could be used in a variety of ways: (1) to predict the effects of the metallic object (e.g., ablation probes) positioning and optimize acquisition and reconstruction parameters for patient imaging through virtual trials, (2) to systematically evaluate and compare vendor MAR techniques, and (3) to create a vast image training library for deep learning-based MAR techniques.

Here, we present our work to develop the metallic object insertion framework, including (1) incorporation of accurate noise and beam hardening models in a projection insertion algorithm, (2) application of the insertion algorithm to IO metallic devices and validation in phantom and patient image data, and (3) demonstration of a proof-of-concept application—generation of training data for convolutional neural network (CNN) based MAR algorithms.

## 2 Methods

### 2.1 Unique Challenges and General Metal Insertion Framework

The proposed framework in this study was based on a clinically validated tool to insert liver lesions, lung nodules, and renal stones into patient projection data from single-energy and dual-energy CT scans.^10^^,^^11^ Compared with previous applications, however, insertion of a metallic object such as an ablation probe presents several significant challenges. First, the volume of interest (VOI) containing the metallic object is much larger than VOIs of liver lesion, lung nodules, or kidney stones. Second, these metallic objects are significantly more attenuating—yielding CT numbers in excess of thousands of HU as compared with ∼50  HU or less for common lesions. As a result, forward projection of a large VOI containing a high-attenuating metallic object more significantly alters the baseline sinogram to which the metallic object is added. These perturbations include effects of the inserted object on quantum noise, electronic noise, and beam hardening, which were inconsequential for lesion insertion tasks and thus ignored in previous models. Finally, the true material compositions and internal geometry of the metallic object vary and are unknown, particularly for interventional probes, thus requiring an investigation into the most appropriate method to digitally model the probes. Overall, insertion of these metallic objects such as ablation probes is therefore significantly more complex and advanced than the insertion of smaller, low attenuating objects.

**Fig. 1:**
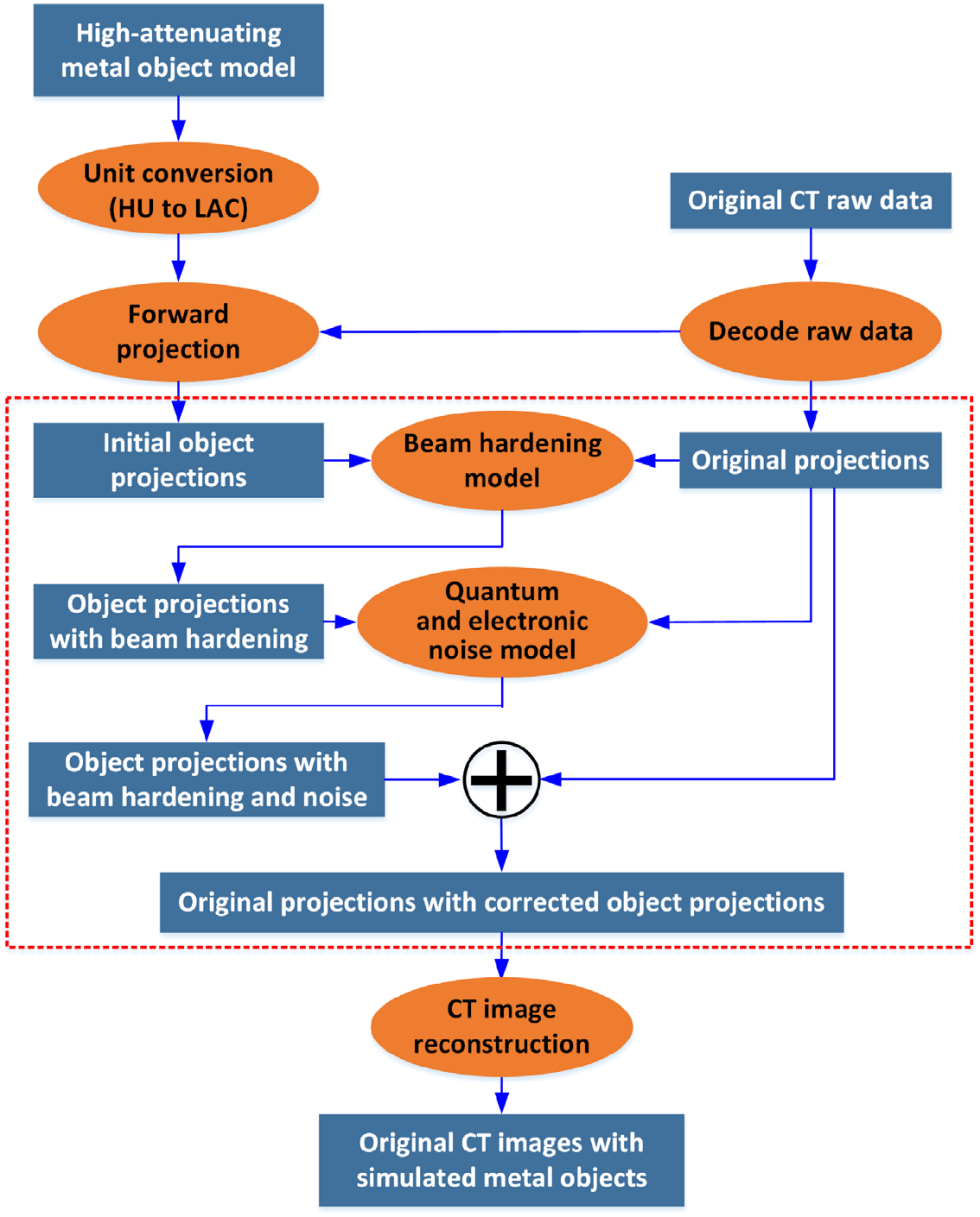
Framework developed for high-attenuating metallic object insertion (HU: Hounsfield Unit; LAC: linear attenuation coefficient).

The proposed metal insertion framework is depicted in Fig. 1. A voxelized high-attenuating metallic object model is forward projected to acquire the initial object projections, which are then manipulated and combined with the original CT projections. The steps manipulating object projection data are highlighted by the dashed red box in Fig. 1 and include models accounting for increased quantum and electronic noise and beam hardening in the presence of the metallic object. Further details about these correction models are explained below. The final combined projections containing the inserted metallic objects are reformatted to match that of commercial CT raw data and reconstructed to yield images simulating the presence of the metallic objects.

#### 2.1.1 Quantum and electronic noise model

An additional noise element is introduced for each line integral intersecting the metallic object $P_{m}(pr,i,j)$, where pr is the index of projection and i and j denote the indices of detector bin along the axial and longitudinal directions, respectively. This noise element $P_{n}(pr,i,j)$ is incorporated to account for the increased contributions of quantum and, in some cases, electronic noise caused by the high attenuation of the metallic object. Specifically, a previously developed noise insertion model^12^ was adapted as

$$P_{n}=\frac{1}{N_{0}\;\exp(-P_{\mathrm{or}})}\sqrt{N_{e}[\exp(2P_{m})-1]+N_{0}\exp(-P_{\mathrm{or}})[\exp(P_{m})-1]}x,$$ (1)

where $N_{0}$ and $N_{e}$ are the incident photon number and the noise-equivalent quanta of the electronic noise floor, respectively; $P_{\mathrm{or}}$ corresponds to the line integral of the original projection; and x is a normally distributed stochastic process with a zero mean and unit variance.

#### 2.1.2 Beam hardening model

The beam hardening model is predicated on parameters derived from the forward projections of the digital metallic object model and the original projections decoded from original CT raw data. The model is comprised of three steps. First, the expected polychromatic x-ray spectrum, spectrum(pr,i,j), that would have been recorded by the x-ray detector (i,j) during projection pr if the metallic object were physically present in the original projections is computed as:

$$\text{spectrum}(i,j)=f({\text{spectrum}}_{\mathrm{or}},\mathrm{WEP}(pr,i,j),\mathrm{DQE}),$$ (2)

where WEP(pr,i,j) denotes the water equivalent path computed for that specific line integral having the same x-ray attenuation as passing through the phantom or patient including the inserted metallic object; ${\text{spectrum}}_{\mathrm{or}}$ represents the original polychromatic x-ray spectrum as it exited the tube housing window, which is identical for all line integrals; and DQE represents the detector quantum efficiency to the incident x-ray beam. The scanner model’s polychromatic x-ray spectrum and detector quantum efficiency were provided by the CT manufacturer.

Second, the mass attenuation coefficient $\mu_{m}(\mathrm{pr},i,j)$ for each line integral intersecting the metallic object based on the new x-ray spectrum is computed as the integration of the original mass attenuation coefficient $\mu_{m_{or},E}$ weighted by the new x-ray spectrum from 10 keV to maximum energy determined by the x-ray tube potential. Since the elemental material for the metallic object may not be available, titanium is used as the reference material for mass attenuation coefficient calculation,

$$\mu_{m}(pr,i,j)=\overset{kV}{\underset{E=10\;\mathrm{keV}}{\sum }}\mu_{m_{or},E}\times \text{spectrum}(pr,i,j).$$ (3)

**Fig. 2:**
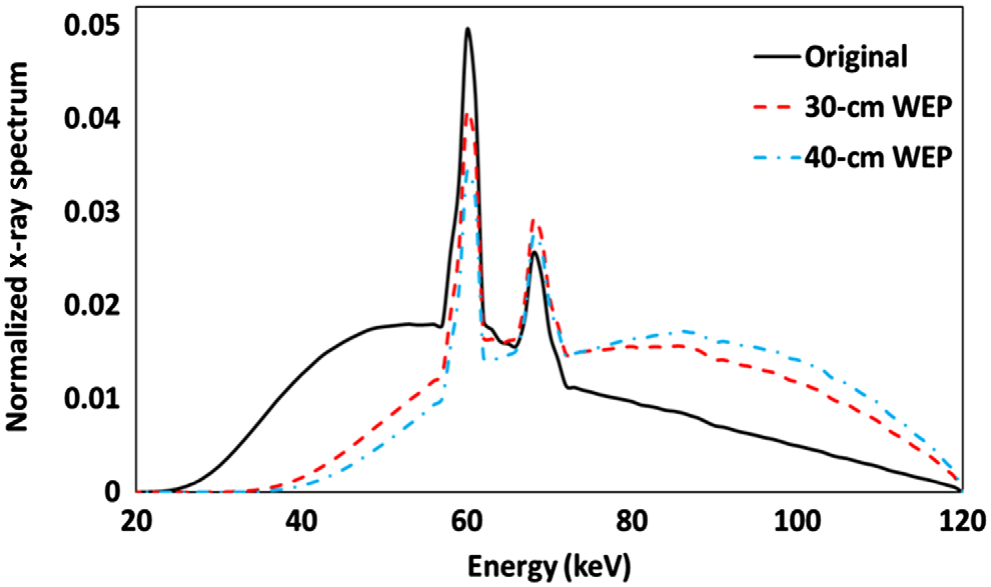
Original polychromatic x-ray spectra exiting from the x-ray tube and recorded spectra being attenuated by 30- and 40-cm water equivalent materials.

A representative plot is shown in Fig. 2 including an original polychromatic x-ray spectrum and two recorded spectra being attenuated by 30- and 40-cm water equivalent materials, respectively. The mass attenuation coefficients, e.g., for titanium, corresponding to the original and the two new spectra were calculated as 0.51, 0.30, and $0.27\;{\mathrm{cm}}^{2}/g$, respectively.

Finally, the metallic object projections are corrected to account for increased beam hardening by scaling each projection intersecting the metal based on the ratio of $\frac{\mu_{m}(pr,i,j)}{\mu_{m_{\mathrm{ave}}}}$, where $\mu_{m_{\mathrm{ave}}}$ is the average mass attenuation coefficient of all line integrals intersecting the metallic object.

#### 2.1.3 Validation of noise and beam hardening models

**Fig. 3:**
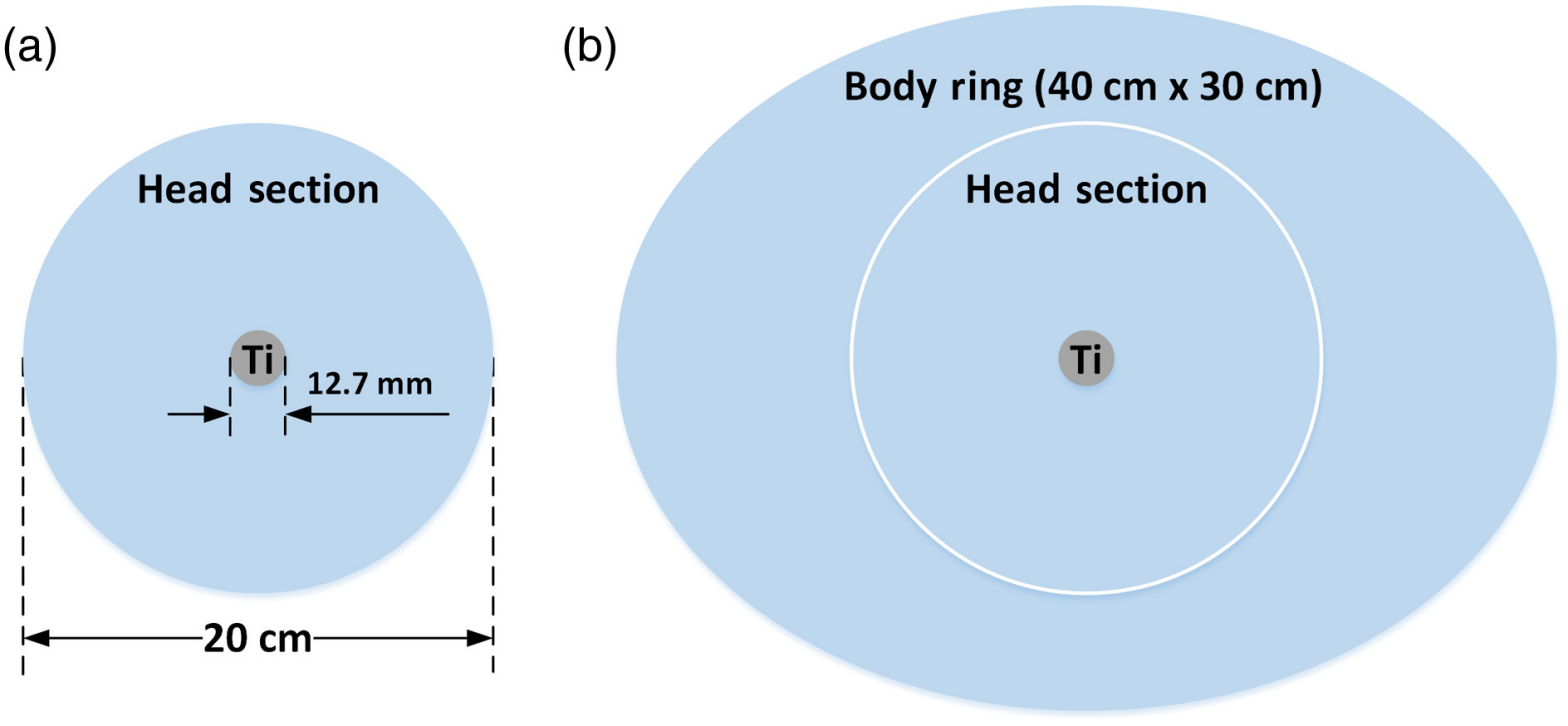
Schematic of (a) head phantom (20-cm diameter) and (b) body phantom (head phantom inserted into elliptical body ring: 30-×40-cm) with solid titanium (Ti) rod inserted in the center hole of the head phantom and all other holes filled with solid water or soft tissue inserts (not shown).

Circular head (20-cm diameter) and elliptical body (30  cm×40  cm) phantoms, both made from water-equivalent material (Gammex Inc., Middletown, Wisconsin, United States), were employed to validate the newly developed physical models in the metal insertion framework. A solid titanium rod (12.7-mm diameter) was inserted into the center hole of the phantom, as illustrated in Fig. 3. All other holes in the phantom were filled with solid-water or soft tissue inserts. All scans were performed with a 128-detector-rows CT scanner (Definition Flash, Siemens Healthcare, Forchheim, Germany).

Two sets of experiments were performed: one set to validate the noise component of the insertion model using the circular head phantom and a second set to validate the beam hardening component of the insertion model using the anthropomorphic body phantom.

In the first experiment, the circular head phantom was selected to minimize beam hardening artifacts stemming from attenuation differences among the nonisotropic projections. The phantom was scanned at 120 kV with 20 and 200 effective mAs, respectively. A separate set of scans was performed with identical configurations but with the titanium rod replaced by a solid-water insert. These data were then used for the artificial insertion of a Ti rod. More specifically, a voxelized cylinder with matching nominal dimensions and material composition, as compared with the real rod, was digitally created and inserted into these projection data, after estimating its CT number attenuation values using a CT-data simulation tool (DRASIM, Siemens Healthcare). To demonstrate the effect of the noise model, CT projections of the Ti rod were combined with the original projections without and with the application of the noise model.

In the second set of experiments, the body phantom was scanned at 120 kV with 200 effective mAs. Similarly, a separate set of scans was performed with identical configurations but with the titanium rod replaced by a solid-water insert for subsequent artificial insertion. The voxelized rod, corrected by the contribution of noise described above, was inserted into the original phantom-only projection data. To demonstrate the effect of the beam hardening model, the Ti rod projections were combined with the original projections both with and without the application of the beam hardening model.

All CT projection data were reconstructed with the following parameters: 1.0/0.5  mm slice-thickness/increment, 512×512 matrix size, medium sharp Br40 kernel, and standard HU scale for comparison. A circular ROI was drawn to quantify the noise levels on the set of head phantom images with the real rod and artificially inserted rod (with/without noise model). The same ROI was placed over 100 consecutive slices, and the final values of noise level were computed as the averages (±SD) from all slices. The projection data acquired from the body phantom with the real and artificial rods were compared. Two rectangular ROIs were placed in both projection images containing the real and simulated rod. One ROI was placed within the projections containing contributions from the rods, and the other ROI was placed in projections of the surrounding water background to quantify the projection values. A difference map was derived as the subtraction between the projection data with the real rod and the artificial rod.

### 2.2 Determination of Optimal Digital Probe Model

**Fig. 4:**
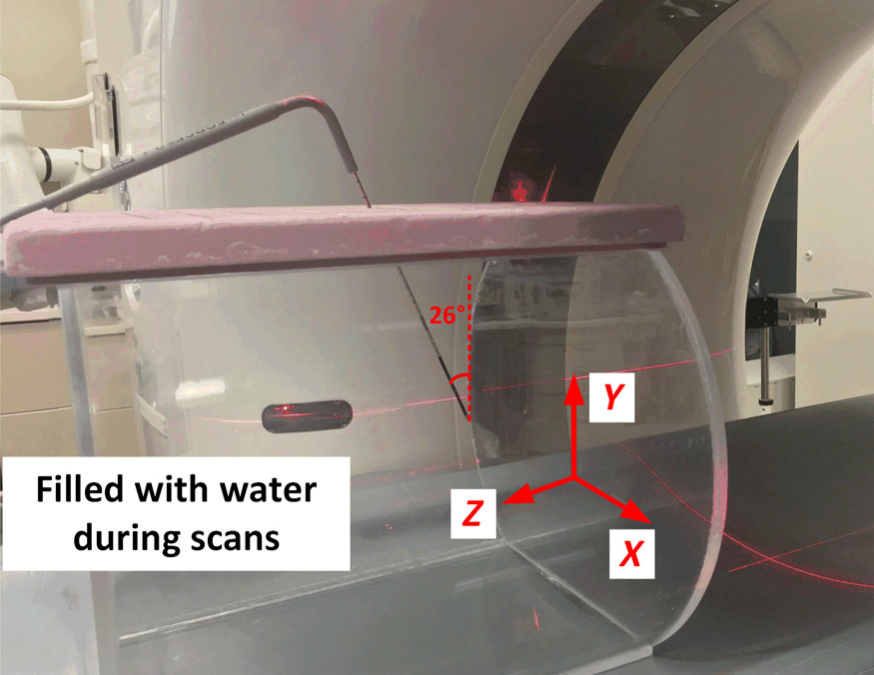
Experimental set-up of probe placement to collect CT images and create a digital probe model

As the true material composition of the probes and exact external and internal dimensions are unknown, probe models in this study were created by segmenting CT images of real probes. A single cryoablation probe (IceForce 2.1 CX, Boston Scientific, Marlborough, Massachusetts, United States), which is routinely used in our IO practice, was investigated to develop the best approach to create accurate digital probe models. First, the probe was imaged with a 128-detector-rows CT scanner (Definition Edge, Siemens Healthcare). The probe was physically inserted into a 35-cm water tank with an oblique angle of ∼26  deg off the X−Y plane to minimize the metal artifacts along the probe shaft, which otherwise limited accurate segmentation of the probe. The probe orientation was depicted in Fig. 4. The probe was then scanned using the acquisition technique for our routine abdomen exam protocol. Essential data acquisition geometry as well as relevant scan parameters are summarized in Table 1. A separate set of scans of the water tank without the probe were performed for subsequent artificial insertion. Three reconstructions were performed: one with routine clinical parameters and two high-resolution reconstructions, with and without an extended HU scale. All reconstruction parameters are summarized in Table 1.

**Table 1:** Summary of essential data acquisition geometry, scan parameters, and reconstruction parameters in water phantom scan.

Scan parameters	Tube voltage (kV)	120		
	Eff. mAs	200		
	Pitch	0.6		
	Rotation time (s)	0.5		
	Collimation (mm)	128×0.6		
	CTDIvol (mGy)	13.5		
Recon parameters	Recon FOV (mm)	200	200	200
	Slice-thickness/increment (mm)	1.0/0.5	0.6/0.2	0.6/0.2
	Matrix size	512×512	1024×1024	1024×1024
	Recon Kernel	Br40	Br70	Br70
	Extended HU scale	No (12 bit)	No (12 bit)	Yes (16 bit)
Insertion parameters	Voxel size of VOI containing probe (mm)	[0.5 0.5 0.5]	[0.2 0.2 0.2]	[0.2 0.2 0.2]

From each set of reconstructed images, the probe was segmented using a customized program (MATLAB 2019b, Mathworks, Natick, Massachusetts, United States) with a combination of adaptive thresholds and connectivity criteria to retain voxels representing probe structures only. To determine the optimal CT reconstruction parameters to create a digital probe model, each segmented probe was directly forward projected and then artificially inserted into a water-only scan. All projections were then reconstructed with identical parameters (slice-thickness/increment: 1.0/0.5  mm; matrix size: 512×512; sharp Br70 kernel) for side-by-side comparison with CT images of the real probe. Multiplanar reformatted (MPR) images were also inspected to qualitatively evaluate the probe and probe-induced artifacts for all three sets of reconstruction parameters. The optimal digital probe model demonstrating the most accurate representation of the probe and probe-induced artifacts was applied for all subsequent phantom and patient studies.

### 2.3 Virtual Probe Library and Graphical User Interface

Digital models for 18 metallic probes that are routinely used in our clinical practice during cryoablation and microwave ablation procedures were developed using probe images from high-resolution reconstructions with an extended HU scale. A digital virtual library was generated to store all of the created probe models. In routine clinical practice, different types and numbers of ablation probes may be physically inserted into patients in a variety of orientations and depths, depending on the specific clinical objectives. An intuitive graphical user interface (GUI: App Designer, MATLAB 2019b, Mathworks, Natick, Massachusetts, United States) was created to facilitate efficient insertion of any number of different probes at arbitrary positions in patient CT data to mimic real and simulate virtual patient procedures. An important feature of this GUI is that the probe(s) can be artificially inserted into patient data in both the image domain and the projection domain at identical positions within the 3D image. Specifically, the images from the image domain insertions are free of artifacts, whereas those from the projection domain insertions contain metal artifacts introduced by the corruption of the projection data and the reconstruction algorithm. The image domain insertions allow the user to quickly review and verify that the desired number and type of the probes were inserted at the prescribed positions, without the need to go through the relatively time-consuming projection domain insertions. Additionally, the generation of these accurately coregistered sets of CT data enables the efficient creation of a vast image library with training targets (artifacts-free image insertions) and inputs (projection insertions with metal artifacts) for the development of deep-learning-based CNN MAR algorithms.

### 2.4 *Ex-Vivo* Validation of Probe Insertion Framework

To validate the accuracy of the developed insertion framework, one cryoablation probe (IceForce 2.1 CX, Boston Scientific) and one microwave ablation probe (Neuwave XT, J&J, New Brunswick, New Jersey, United States) were positioned such that they were coplanar with the imaging plane to provide a near-worst-case scenario for probe-induced artifacts. The water phantom was scanned using the same acquisition technique described in Table 1 with and without the two probes. The proper digital probe models were selected, translated, and rotated to match the position and orientation of the real probes and inserted into the water-only projection data. Both sets of projection data with real and artificially inserted probes were reconstructed with identical parameters (slice-thickness/increment: 1.0/0.5  mm; matrix size: 512×512; medium sharp Br40 kernel) for qualitative comparison.

To quantify the volume affected by the probe-induced artifacts, histogram distributions of all of the voxels with HU values <−75 or [75, 500] were analyzed. Thresholds of −75 and 75 HU were used to define the artifactual signal (±3 standard deviations of the water-only histogram), and the upper 500 HU limit was set to exclude voxels corresponding to the metallic probe.

### 2.5 Demonstration of Probe Insertion with Patient Data

With the approval of our institutional review board, the metallic object insertion framework was applied to CT projection data from patient ablation procedures. Two percutaneous cryoablation cases were used for demonstration.

In the first case, four cryoablation probes were employed to treat two small masses contained in a solitary kidney. Multiple helical CT scans, referred to as “probe” scans, were performed during the procedure to confirm the probe positions as they were systematically inserted by the radiologist. Projection data from the first two probe scans were used to validate the insertion model. Specifically, the first probe scan was performed after placing the first three probes and the second probe scan was executed after all four probes were placed. The proper digital probe model from the probe library was selected to match the type, position, and orientation of the fourth probe that was inserted during the procedure and visible in the second probe scan only. This digital probe was then inserted into projections from the first probe scan. In the reconstructed CT volumes, the artificially inserted probe was qualitatively compared with the corresponding real probe in the second probe scan and the other three real probes contained in the same CT volume.

Similar to the first case, four cryoablation probes were placed to treat a right renal mass on a second patient. Multiple probe scans were performed to confirm probe placement accuracy during the procedure, and projection data from the first two probe scans were used to validate the insertion model. The first scan was performed after a single probe insertion, and the second scan was performed after inserting the remaining three probes. The final three probes placed during the procedure were selected from the digital probe library and artificially inserted into the projections of the first scan. In the reconstructed CT volumes, three artificially inserted probes were qualitatively compared with the corresponding real probes in the second probe scan and another real probe contained in the same CT volume.

Essential data acquisition geometry, scan parameters, and reconstruction parameters for these two patient cases are summarized in Table 2 (first two patient cases).

**Table 2:** Summary of essential data acquisition geometry, scan parameters, and reconstruction parameters of selected patient cases.

Percutaneous cryoablation cases		First patient case	Second patient case	Third patient case
Type of scans		Probe scan	Probe scan	Planning scan
Scan parameters	Tube voltage (kV)	140		100
	AEC	ON		
	Quality reference mAs	68		275
	Pitch	0.6		0.8
	Rotation time (s)	0.5		
	Collimation (mm)	128×0.6		
	CTDIvol (mGy)	11.4	24.37	15.0
Recon parameters	Recon FOV (mm)	200	350	380
	Slice-thickness/increment (mm)	3.0/3.0		
	Matrix size	512×512		
	Recon Kernel	Br40		
	HU scale	12 bits		

### 2.6 Demonstration of a Proof-of-Concept Application: CNN Training Images

As described in Sec. 2.3, the probe(s) can be artificially inserted into patient data in both the image domain and the projection domain at identical positions within the 3D image, and therefore, the generated CT image datasets are inherently co-registered as training input/target pairs for the development of deep-learning-based CNN MAR algorithms. To further demonstrate the potential of the developed framework for this application with a more complicated scenario in clinical practice, one percutaneous kidney cryoablation case was selected and both image and projection data were exported from the planning CT scan. Four cryoablation probes of two different types (IceForce 2.1 CX, Boston Scientific; Endocare V-Probe, Varian, Palo Alto, California, United States) were inserted in the image domain and the projection domain. Essential data acquisition geometry, scan parameters, and reconstruction parameters for the above patient case are summarized in Table 2 (third patient case).

## 3 Results

### 3.1 Validation of Noise and Beam Hardening Models

**Fig. 5:**
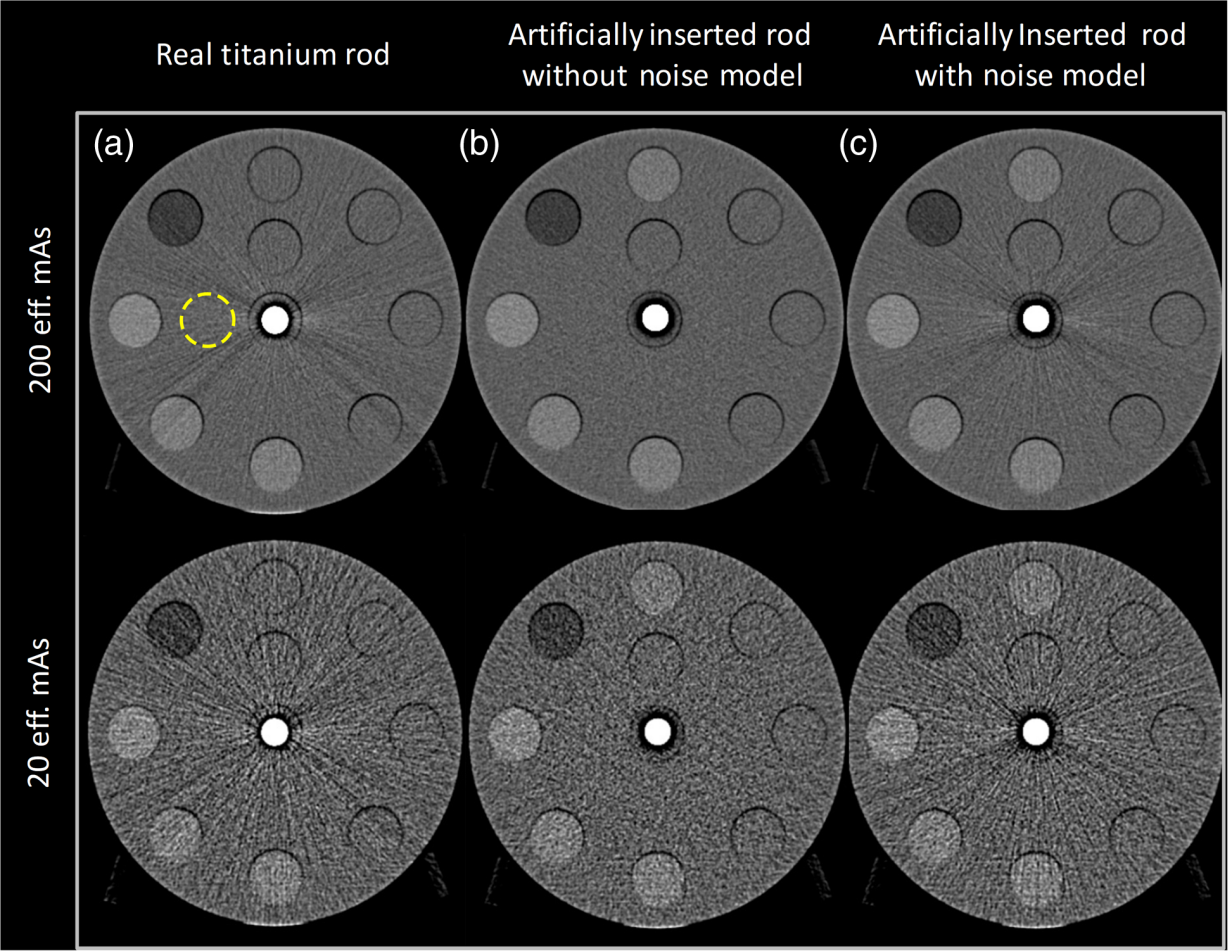
Comparison of head phantom images with the real titanium rod and the artificially inserted rod without/with noise model at two mAs levels: (a) real titanium rod, (b) simulated rod without noise model, and (c) simulated rod with noise model. Top: 200 effective mAs. Bottom: 20 effective mAs.

Figure 5(a) depicts the head phantom images reconstructed from projections containing the real and artificially inserted titanium rods; Fig. 5(b) shows without noise model; Fig. 5(c) shows with noise model acquired with the two different effective mAs levels (top row: 200 eff. mAs; bottom row: 20 eff. mAs). Incorporating the noise model yielded a more similar appearance between the images of the real and simulated rods at both extremes of radiation doses, indicating that both quantum and electronic noise are properly modeled.

The ROI that was drawn to quantify the noise levels are displayed in Fig. 5, and the measured values are 41.06±1.48 (real rod), 35.37±1.37 (simulated rod without noise model), and 39.78±1.50 (simulated rod with noise model) for 20 eff. mAs acquisitions and 14.32±0.68 (real rod), 11.53±0.43 (simulated rod without noise model), and 13.21±0.53 (simulated rod with noise model) for 200 eff. mAs acquisitions.

**Fig. 6:**
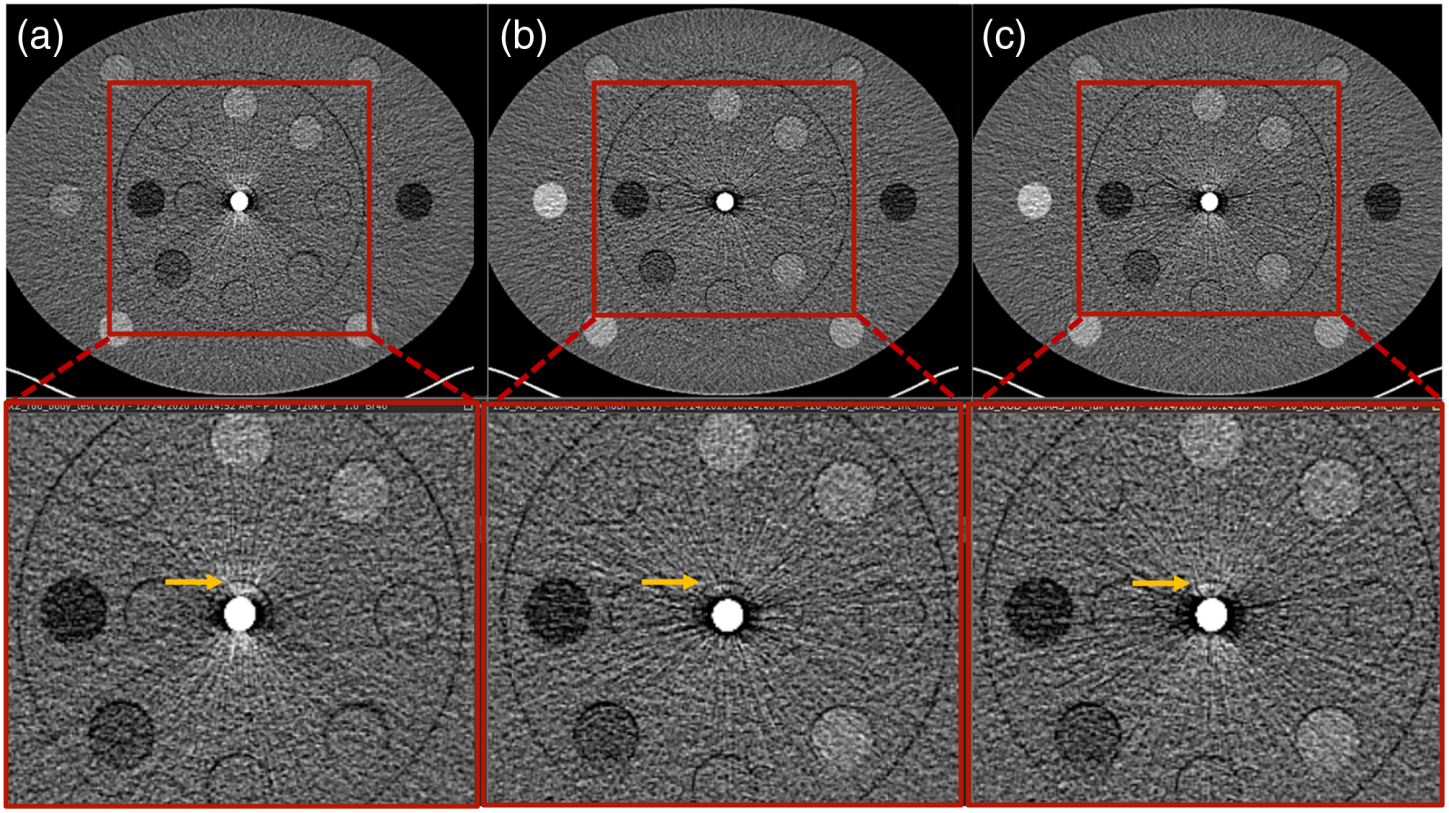
Comparison of body phantom images with the real titanium rod and the simulated titanium rod without/with beam hardening model: (a) real titanium rod, (b) simulated rod without beam hardening model, and (c) simulated rod with beam hardening model.

Figure 6(a) depicts the body phantom images reconstructed from projections containing the real and artificially inserted Ti rods; Fig. 6(b) shows without beam hardening model; Fig. 6(c) shows with beam hardening model. Without the beam hardening model, the bright blooming artifacts stemming from the rod along the anterior–posterior direction were absent; however, they were well-replicated in images of the artificially inserted rod that included the beam hardening model, indicating that beam hardening is adequately modeled.

**Fig. 7:**
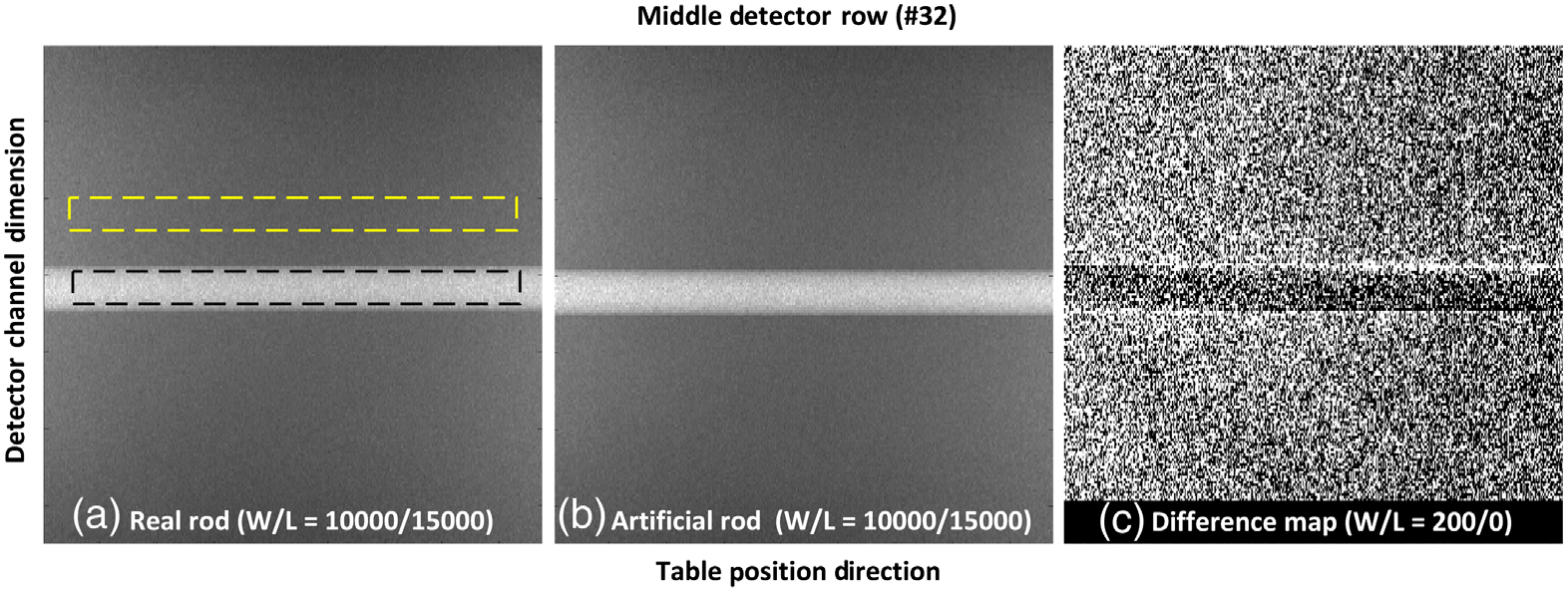
Comparison of projection data acquired with (a) real rod, (b) artificial rod, and (c) difference between real and artificial rods. Note that the middle detector row (i.e., 32) was selected for this comparison (W/L = window/level).

The projection data acquired with real and artificial rods are compared in Fig. 7. Without loss of the generality, the projection data corresponding to the middle detector row (i.e., row number=32) were selected and cropped to reveal details of the rods. The ROIs placed to quantify the projection values (mean±SD) were shown in Fig. 7(a). The measured values were 17,678±542 and 17,787±639 for the real and artificial rods, respectively. For reference, the surrounding water region had measured values of 13,979±427 and 13,960±402, respectively. The difference map (projection data with real rod–projection data with artificial rod) was displayed in Fig. 7(c). Note that a much narrower window of 200 was selected compared with that used to display the original projection data (W=10,000). The slight misalignment of the rods manifested on the difference map was caused by imperfectly aligned insertion positions, as well as differences in the x-ray tube starting angles between the two acquisitions.

### 3.2 Determination of Optimal Digital Probe Model

**Fig. 8:**
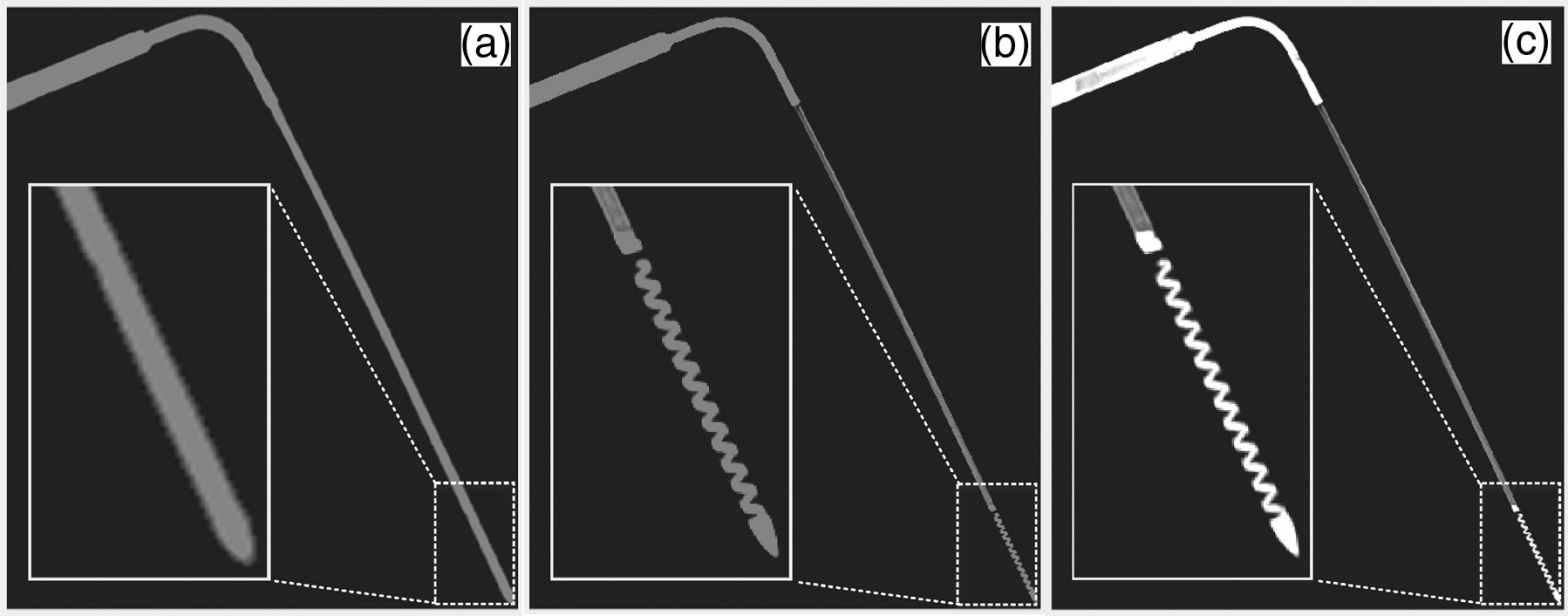
Segmented probes from images with (a) routine reconstruction, (b) high-resolution reconstruction with a standard HU scale, and (c) high-resolution reconstruction with an extended HU scale; window/level=10,000/2000  HU.

Figure 8 shows the segmented probes from images reconstructed with routine parameters and high-resolution parameters without and with the extended HU scale. Compared with the images reconstructed with routine parameters, the visualization of the internal helically coiled wire at the distal end of the probe was much improved in the images reconstructed with high-resolution parameters due to less partial voluming. With the extended HU scale, the high-resolution images could further provide more density distribution differences among different components of the probe, e.g., relatively higher attenuation of the probe’s distal end and a handle and relatively lower attenuation of the probe shaft. The higher fidelity images of the probe reconstructed with high-resolution parameters and extended HU scale enables more accurate modeling of the probe in the subsequent forward projection.

**Fig. 9:**
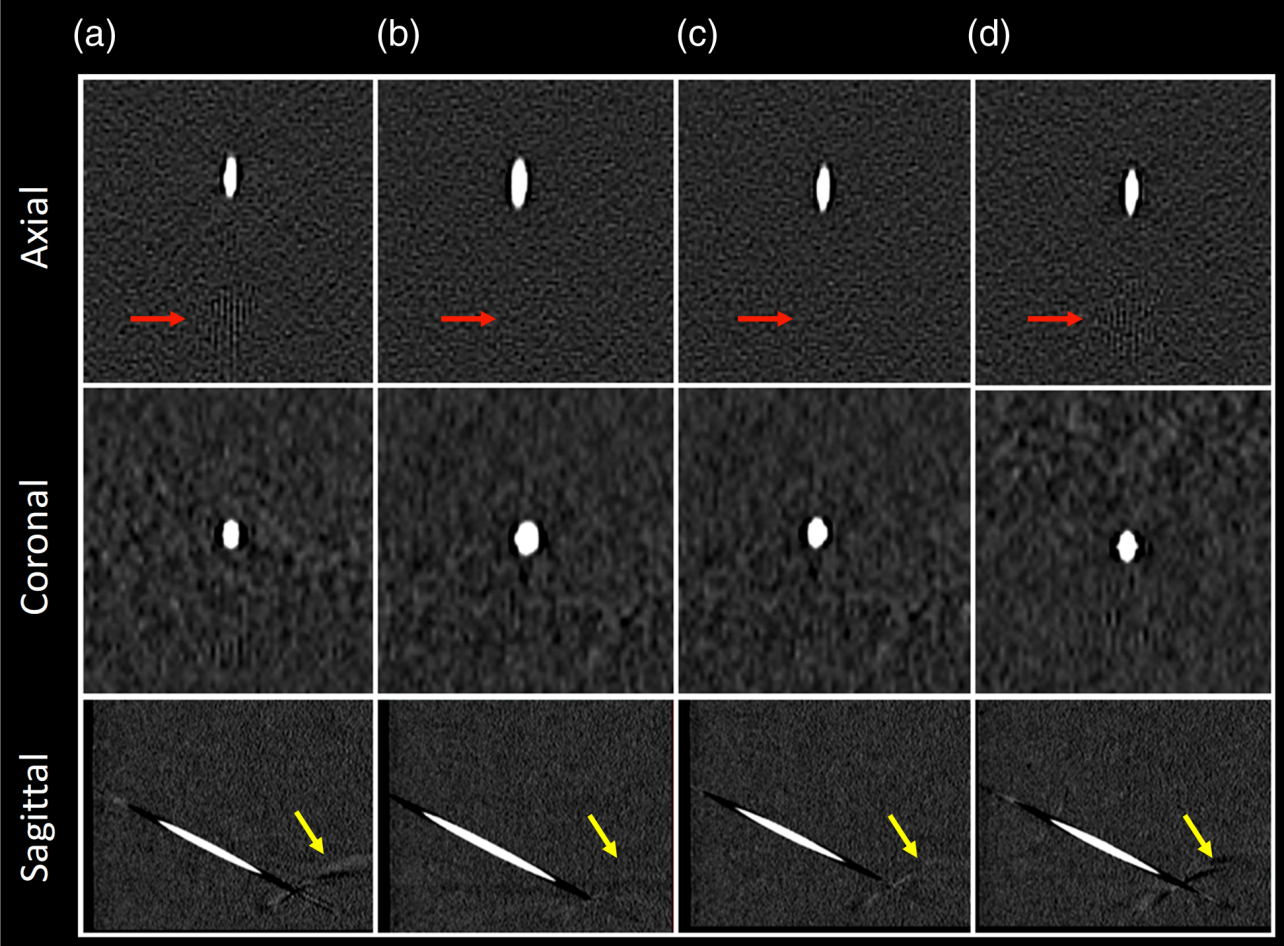
(a) Real probe. (b–d) Artificially inserted probes from probe model created with (b) routine reconstruction, (c) high-resolution reconstruction, and (d) high-resolution reconstruction with an extended HU scale, respectively; from top to bottom: axial, coronal, and sagittal planes (W/L=1500/500  HU).

Figure 9 compares MPR images of the real probe (first column) with those of the artificially inserted probes (second through fourth columns) for all three reconstruction settings. The higher fidelity of the digital model obtained from the high-resolution and extended HU scale reconstruction appears necessary to reproduce the realistic probe and probe-induced artifactual signals, particularly those stemming from small, high-attenuating substructures, as shown in Fig. 9 (red and yellow arrows). This optimal reconstruction setting was, therefore, used to create all-digital probe models for future probe insertions with phantom and patient data.

### 3.3 Virtual Probe Library and Graphical User Interface

**Fig. 10:**
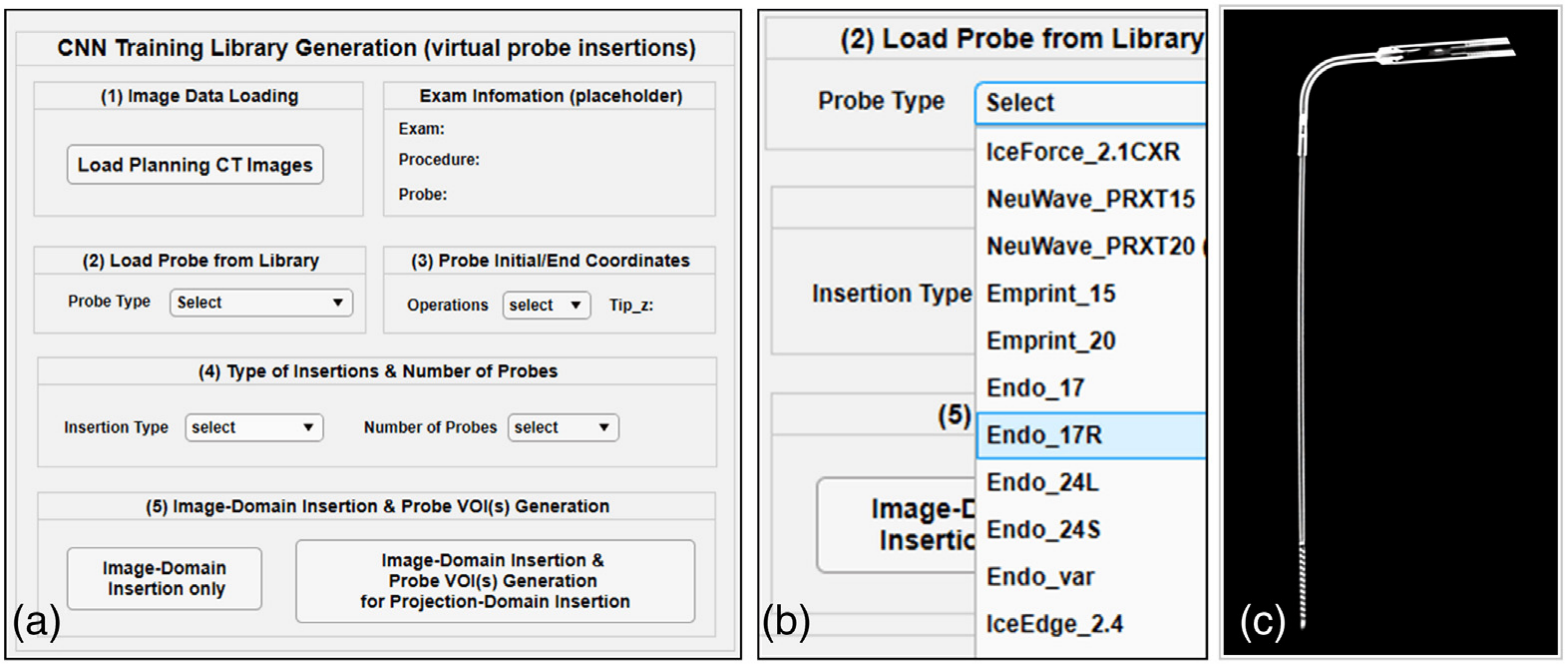
(a) GUI designed for efficient probe insertion and CNN training data generation; (b) drop down menu listing a sample of the list of available probes in the library; (c) one example of the digital probe model (IceForce 2.1 CX).

Figure 10(a) shows the GUI that was developed for efficient probe insertions. Upon loading a CT image dataset (with or without metallic probes already present), the user selects the digital probe model from the library. A partial list of available probes is shown in Fig. 10(b), and one example probe model is shown in Fig. 10(c). The user then determines the desired tip location(s) and the orientation(s) of the inserted probe(s). If one or more probes are to be inserted at different orientations, the user can choose between different insertion types/conditions (e.g., single probe only, multiple probes converging to a single region, or multiple probes in parallel). Finally, the user can choose to perform image domain insertions only–to review and verify that all of the required information was correct–or to combine it to the generation of a probe VOI that contains all of the parameters required to perform projection domain insertion.

### 3.4 *Ex-Vivo* Validation of Probe Insertion Framework

**Fig. 11:**
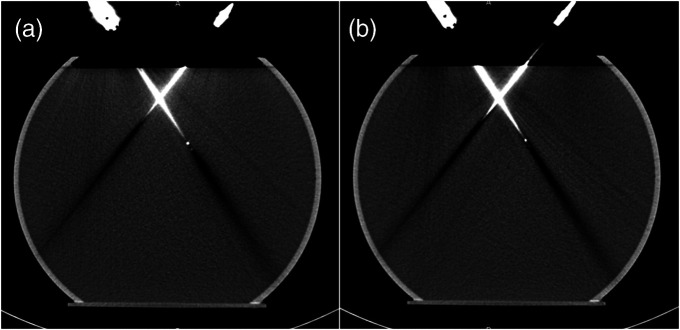
Comparison of probes placed coplanar with the imaging plane: (a) real probes and (b) artificially inserted probes (W/L=400/40  HU). Notably, to reveal two probes and their associated artifacts in a single image, the reconstructed CT images (slice thickness: 1.0 mm) were reformatted to 5.0 mm.

**Fig. 12:**
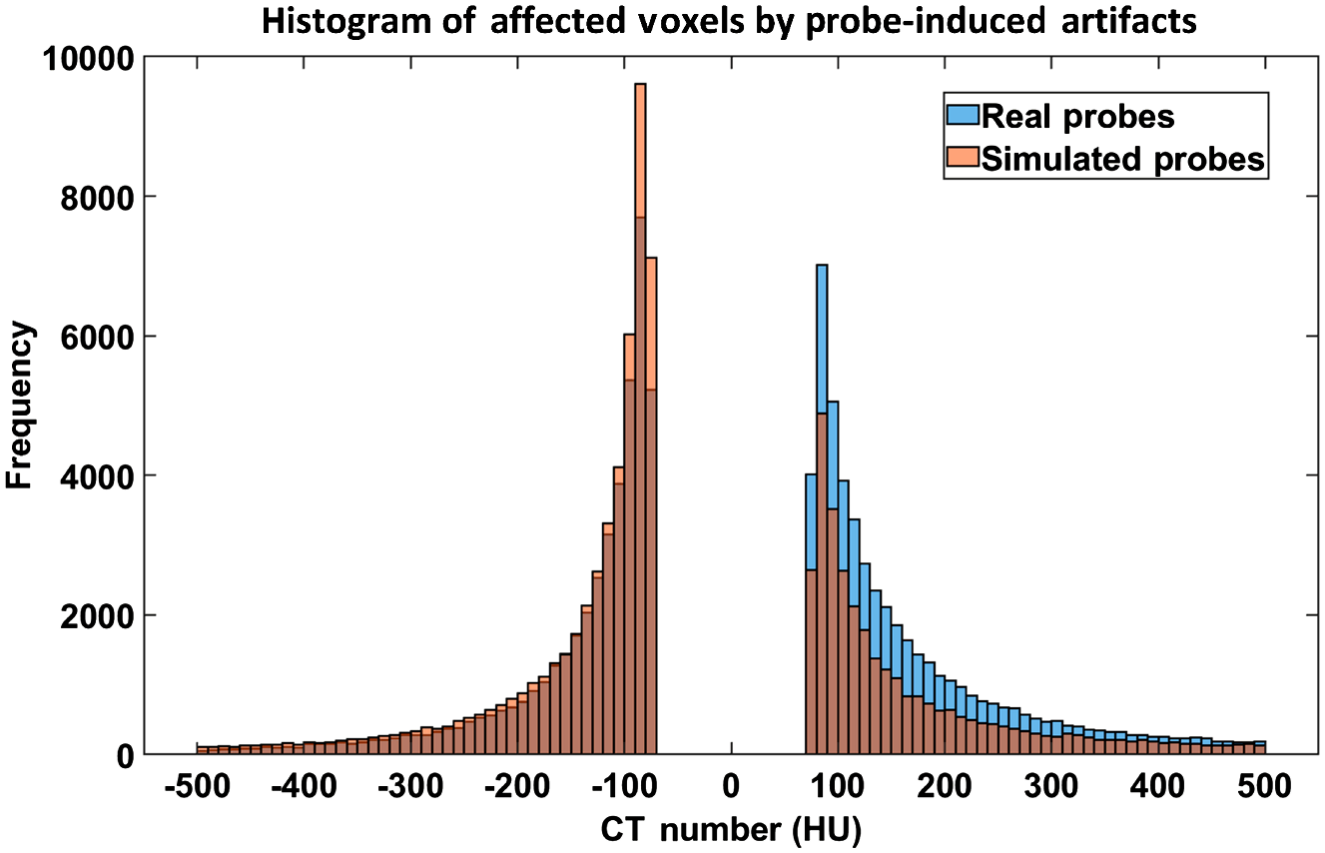
Comparison of histogram distributions (HU values <−75  HU or within [75, 500 HU]) of the volume affected by the probe-induced artifacts for real and artificially inserted probes.

Figures 11(a) and 11(b) show water phantom images with two real probes compared with images of the same probes artificially inserted in a water-only phantom. Artifacts induced by each probe are accurately reproduced. Histogram distributions of the voxels within the volume affected by the probe-induced artifacts are plotted and compared in Fig. 12. The low value histograms (<−75  HU) corresponding to the dark streaking artifacts along the probe shaft were well matched, whereas the high-value histograms ([75, 500 HU]) corresponding to the blooming artifacts perpendicular to the probe shaft were slightly lower for the artificially inserted probes compared with real probes.

### 3.5 Demonstration of Probe Insertion into Patient Data

**Fig. 13:**
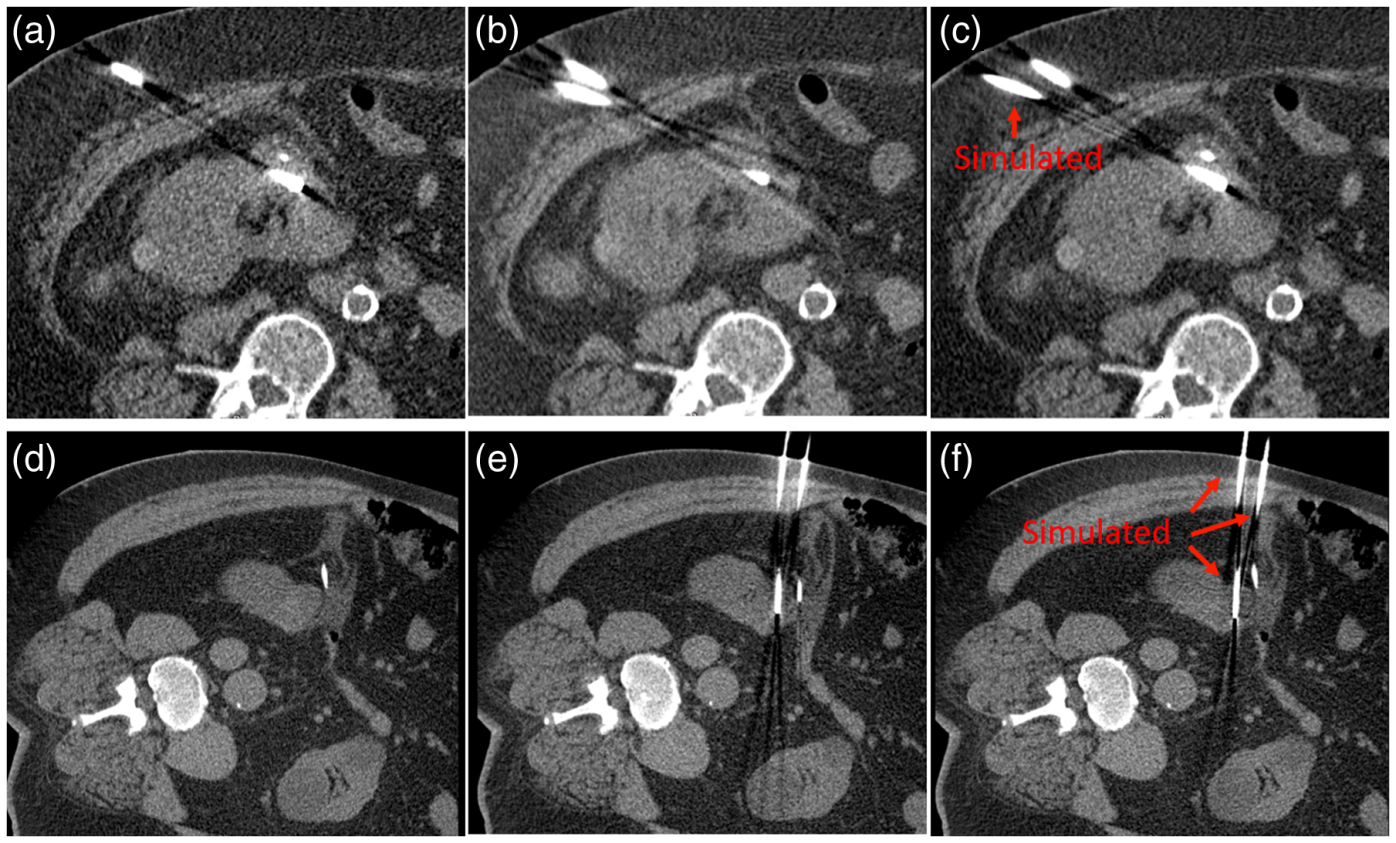
Patient case 1: (a) first scan with three real probes, (b) second scan with four real probes, and (c) first scan with three real probes and one artificially inserted probe; patient case 2: (d) first scan with one real probe, (e) second scan with four real probes, and (f) first scan with one real probe and three artificially inserted probes (W/L=400/40  HU).

Figure 13 shows probe scan images for two patient cases: (a) and (d) first probe scans with (a) three real probes and (d) one real probe, (b) and (e) second probe scans with the addition of one and three real probes, respectively, and (c) and (f) first probe scans with the addition of three stimulated probes and one simulated probe, respectively. The relative movement of the patient’s organs and the inserted probes between two scans prevented quantitative comparison. As shown in Figs. 13(c) and 13(f), however, the simulated probes are indistinguishable from real ones contained in the same CT image. In particular, in the second patient case where the relative probe locations are more similar, the artifact appearance between real (e) and simulated (f) probes is virtually identical.

### 3.6 Demonstration of Generating Training Images for CNN MARIO

**Fig. 14:**
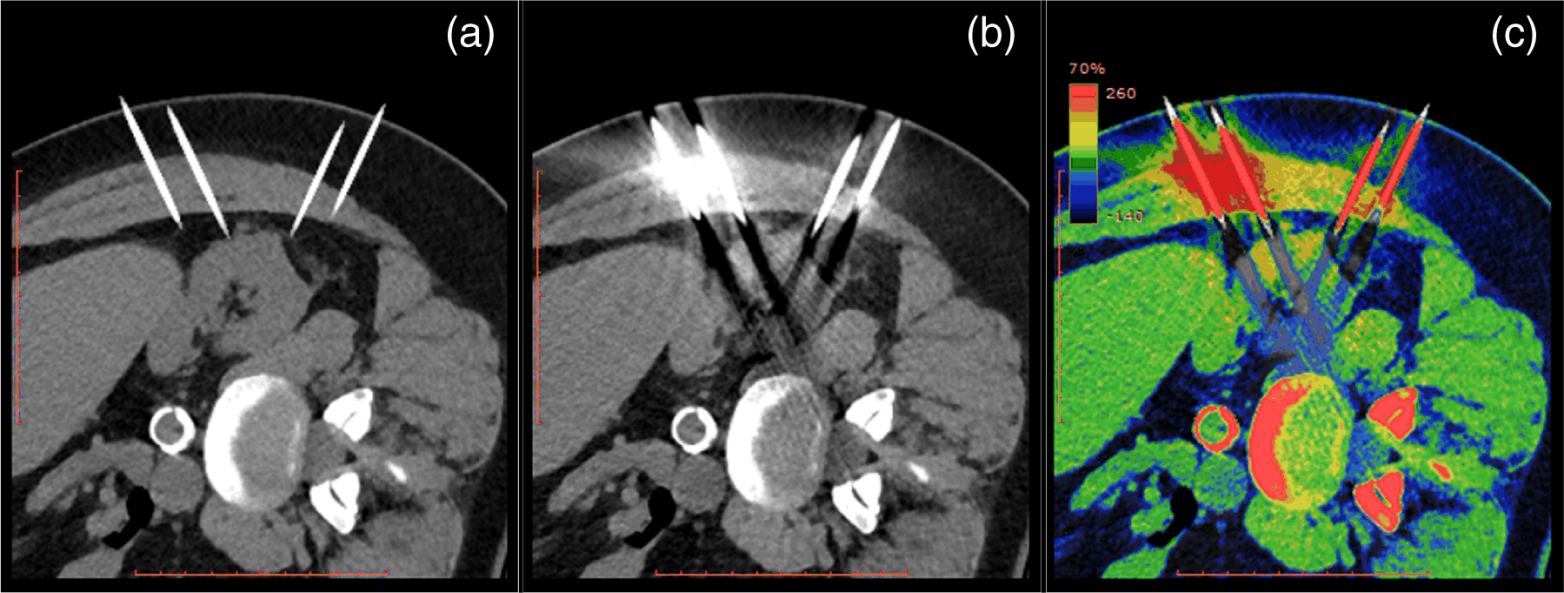
Demonstration of (a) image domain insertion with probes only and free of artifacts, and (b) projection domain insertions and (c) pseudocolor overlay of the projection domain insertion overlapping the image domain insertion image showing exact alignment of the probes (W/L=400/40  HU). Perfect registration between image and projection domain metallic object insertion is essential for this framework to be able to generate the required data to train CNN-based MAR algorithms.

Despite accurate simulation of scanner projection geometry, precise coregistration of image domain and project domain probe insertions is not guaranteed. For these data to be effectively used for training CNN algorithms, accurate coregistration is a requirement. Figure 14 shows (a) image domain and (b) projection domain metal probe insertions in the planning CT scan. Excellent spatial coregistration between the input (images with artifact) and target (artifact-free images) images generated in the projection domain and image domain with the developed metal insertion framework is demonstrated in the pseudocolor overlay image in Fig. 14(c). Of note, the probes inserted in the image domain appear longer than the corresponding probes in the projection domain due to the metal artifacts obscuring the true length of the probe captured in the image.

## 4 Discussion and Conclusions

In this study, a framework to accurately simulate the presence of metal artifacts in CT images by artificially inserting a metallic object in the projection domain was developed and tailored for CT-guided percutaneous ablation procedures. As part of the developed framework, an intuitive GUI was built to facilitate efficient insertion of any number of different probes at arbitrary positions in patient CT data, to mimic both real and simulated patient procedures. Compared with prior work on lesion insertion in CT projections, the developed framework explicitly accounts for distortions of the CT projection data introduced by the high attenuating metallic objects. The developed models for quantum and electronic noise, as well as beam hardening, were successfully validated with phantom experiments. Further, a digital probe model derived from high-resolution reconstruction with an extended HU scale was shown to more accurately replicate the metallic probe and artifacts compared with those generated from other digital probe models. The feasibility of the developed ablation probe insertion framework was successfully demonstrated with phantom data and an arbitrary arrangement of probes. Clinical applicability of the insertion model was demonstrated with patient data, despite differences in some of the essential scan parameters of the patient data, such as tube voltage, radiation dose, and automatic exposure control (AEC) setting. Using the designed GUI, creation of unique pairs of images through the projection (images with artifact) and image (artifact-free images) domain insertions could be readily generated, including those for different ablation procedures with various combinations of probe types, numbers, and orientations.

Importantly, insertion of the metallic object in the projection domain rather than in the image domain was vital to accurately replicate artifact appearance.^13^ Another benefit of projection domain insertions is the ability to train CNN MAR algorithms in the image domain, projection domain, or dual-domain (projection domain+image domain).^14^^,^^15^ For instance, one could use our framework to generate registered projection data containing metal at an arbitrary location with and without the corruptions modeled in our work (i.e., beam hardening, quantum, and electronic noise) and train a CNN model to identify and correct these corruptions prior to reconstruction. Zhang and Yu^16^ recently introduced a CNN algorithm dedicated to MAR. Although not tailored exclusively to CT-guided procedures, their work also relied on the insertion of metallic objects in the CT projection domain. However, there are a few substantial differences between our approach and their work. First, they used generic forward- and back-projection algorithms to obtain metal and metal-free images. The appearance of metal artifacts is significantly affected by the vendor-specific acquisition and reconstruction parameters used to create CT images, which are typically proprietary. Our framework inserted metallic objects directly into the acquired CT projection data, and the combined projections were reconstructed at the CT scanner console using the same algorithms as those used for clinical patient data. Additionally, our insertion model directly and explicitly accounted for the effects of AEC, bowtie filter, and quantum and electronic noise.^12^ The projection file contains the mA value used for each projection and thus inherently incorporates AEC. To account for the bowtie filter, air calibrations were performed to estimate photon flux on each detector at a given mA. More details regarding how the effects of AEC and the bowtie filter were incorporated into the noise model were reported previously.^12^ Finally, we developed a strategy to generate device-specific digital models that possessed internal structures that significantly impact artifact generation, whereas the cited work relied solely on arbitrary shapes similar to the metallic objects being simulated.

Despite successfully demonstrating accurate metallic object and artifact insertion, this study was limited in several ways. First, some effects were not considered or modeled in the development of the metal insertion framework; these include the finite focal spot size (assumed a point source) and perturbations from the inserted objects on x-ray scatter distribution. Compton scatter is a significant contributor to the formation and appearance of metal artifacts and as such cannot be fully ignored.^17^^,^^18^ Notably, Compton scatter from the patient tissue is indeed present in our projections as those projections originate from actual x-ray exposures during CT exams. However, due to the relatively small cross-section and the high atomic number of the metallic probes, it is believed that the contribution of perturbations from the inserted objects on the scatter distribution are negligible in our model. This assertion appears to be validated by the results, particularly in Figs. 13(c) and 13(f), where the simulated probes are indistinguishable from real ones contained in the same CT image. The developed framework may need to be revisited if the amount of inserted metal increased (e.g., in the case of orthopedic implants). Second, there was a lack of material composition information for probes, and the probes were therefore segmented and digitized from phantom scans with HU values corresponding to a specific x-ray tube potential (i.e., 120 kV). When inserting the probe models into the projection data of a scan with a different x-ray tube potential, the difference in HU values was not calibrated. Third, k-edge effects were not included in the beam hardening model, for in our applications, the actual elemental material of the metallic probes may not be available, which is why titanium was used as the reference material. However, the presented results demonstrated that these effects played a marginal role in artifact appearance and may thus be considered second-order effects. Fourth, slight misregistration between the real probes and the artificially inserted probes was noticed in the patient data, and this was in part due to the inherent accuracy of the 3D volume rotation algorithm with a pixelized thin object and in part due to the relative movement of the patient’s organs and the inserted probes. However, applications of metallic object insertion in CT images are not expected to require a perfect registration with a reference, metal-corrupted CT dataset. Additionally, minor residual differences in artifact appearance were noticed for the inserted probes in phantoms and patient data. They were likely attributable to slight position discrepancies as well as these nonmodeled second-order effects. Finally, this framework was developed on a single CT scanner model; however, the approach is effectively vendor-neutral and can be applied with proper access to vendor projection data.

The ability to accurately insert metallic ablation probes at arbitrarily locations within CT patient data with realistic artifact replication may benefit many clinical applications in interventional CT and in CT imaging in general. For example, it would enable virtual clinical trials within IO, such as one to compare radiologist’s confidence in probe positioning and treatment planning as a function of trajectory or probe type. Another application would be to optimize CT data acquisition and reconstruction parameters for CT-guided ablation protocols. Finally, as had been demonstrated in the current study, it would greatly facilitate the development of novel, dedicated MAR algorithms through the efficient creation of reference libraries that could be used to train a deep CNN to recognize and remove metal artifacts while maintaining the underlying patient anatomy. More comprehensive investigations of the developed metal insertion framework and its clinical applications are underway.
